# Automated MRI-based quantification of posterior ocular globe flattening and recovery after long-duration spaceflight

**DOI:** 10.1038/s41433-021-01408-1

**Published:** 2021-01-29

**Authors:** Stuart H. Sater, Austin M. Sass, Jesse J. Rohr, Karina Marshall-Goebel, Robert J. Ploutz-Snyder, C. Ross Ethier, Michael B. Stenger, Larry A. Kramer, Bryn A. Martin, Brandon R. Macias

**Affiliations:** 1 Alcyone Therapeutics Inc., Lowell, MA USA; 2 Neurophysiological Imaging and Modeling Laboratory, University of Idaho, Moscow, ID USA; 3 KBR, Houston, TX USA; 4 Applied Biostatistics Laboratory, School of Nursing, University of Michigan, Ann Arbor, MI USA; 5 Wallace H. Coulter Department of Biomedical Engineering, Georgia Institute of Technology and Emory University, Atlanta, GA USA; 6 Cardiovascular and Vision Laboratory, Johnson Space Center, National Aeronautics and Space Administration, Houston, TX USA; 7 Department of Diagnostic and Interventional Imaging, University of Texas Health Science Center at Houston, McGovern Medical School, Houston, TX USA

**Keywords:** Eye manifestations, Visual system, Physiology, Biological techniques

## Abstract

**Background/Objectives:**

Spaceflight associated neuro-ocular syndrome (SANS), a health risk related to long-duration spaceflight, is hypothesized to result from a headward fluid shift that occurs with the loss of hydrostatic pressure gradients in weightlessness. Shifts in the vascular and cerebrospinal fluid compartments alter the mechanical forces at the posterior eye and lead to flattening of the posterior ocular globe. The goal of the present study was to develop a method to quantify globe flattening observed by magnetic resonance imaging after spaceflight.

**Subjects/Methods:**

Volumetric displacement of the posterior globe was quantified in 10 astronauts at 5 time points after spaceflight missions of ~6 months.

**Results:**

Mean globe volumetric displacement was 9.88 mm^3^ (95% CI 4.56–15.19 mm^3^, *p* < 0.001) on the first day of assessment after the mission (R[return]+ 1 day); 9.00 mm^3^ (95% CI 3.73–14.27 mm^3^, *p* = 0.001) at R + 30 days; 6.53 mm^3^ (95% CI 1.24–11.83 mm^3^, *p* < 0.05) at R + 90 days; 4.45 mm^3^ (95% CI −0.96 to 9.86 mm^3^, *p* = 0.12) at R + 180 days; and 7.21 mm^3^ (95% CI 1.82–12.60 mm^3^, *p* < 0.01) at R + 360 days.

**Conclusions:**

There was a consistent inward displacement of the globe at the optic nerve, which had only partially resolved 1 year after landing. More pronounced globe flattening has been observed in previous studies of astronauts; however, those observations lacked quantitative measures and were subjective in nature. The novel automated method described here allows for detailed quantification of structural changes in the posterior globe that may lead to an improved understanding of SANS.

## Introduction

More than half of the astronauts who participate in long-duration spaceflight missions present with neuro-ocular changes that can affect visual acuity. These changes can include choroidal folds, optic disc oedema, hyperopic shifts, and posterior globe flattening, and these signs are associated with spaceflight associated neuro-ocular syndrome (SANS) [1–4]. The severity of visual disturbance reported by astronauts appears to increase with the duration of spaceflight, and some of these disturbances are unresolved after return to Earth [5, 6]. Globe flattening occurs when the convexity of the posterior aspect of the sclera is reduced and the axial length of the globe decreases, which drives a hyperopic shift in refractive error.

Mader et al. described a case study of one astronaut with globe flattening associated with spaceflight [4]. In addition, Kramer et al. observed globe flattening in 7 subjects in a cohort of 27 astronauts [1]. However, in both of these studies, globe flattening was determined subjectively and retrospectively, with limited preflight baseline scans available for comparison. Here we report the first prospective quantitative analysis of spaceflight-induced globe flattening determined by magnetic resonance imaging (MRI).

A leading hypothesis for the underlying pathophysiology of SANS, including globe flattening, is a sustained cephalad fluid shift during long-duration exposure to microgravity [7]. Because the intracranial and retro-orbital cerebrospinal fluid (CSF) spaces communicate, changes in intracranial pressure (ICP) may cause a reversal of the translaminar pressure difference (TLPD) at the posterior eye, i.e., ICP can become greater than intraocular pressure. Such a reversal could cause the sclera to become flattened, as seen in patients with idiopathic intracranial hypertension (IIH) [8–11].

The aim of this study was to quantify spaceflight-induced volume displacement of the posterior ocular globe and to assess the recovery profile for 1 year after flight in an astronaut cohort. To accomplish this, we developed an automated method to perform segmentation of the ocular globe from MR images. The resulting segmentations were parameterized to compare posterior globe shape to preflight (baseline) shape.

## Materials and methods

### Study participants

This study included ten astronauts who participated in missions on the International Space Station (*n* = 20 eyes, mean ± standard deviation (SD) age: 42.9 ± 5.6 years, body mass index: 24.0 ± 1.8 kg/m^2^, and flight duration: 167 ± 17 days). This study’s *n* was limited by the few existing long-duration spaceflight astronauts. Astronauts were scanned 508 ± 230 days before flight, as well as at 4 ± 2 days (R + 1), 31 ± 5 days (R + 30), 101 ± 14 days (R + 90), 196 ± 34 days (R + 180), and 359 ± 19 days (R + 360 days) postflight, allowing for a longitudinal analysis of postflight recovery. Data from 22 time points that were collected from 8 different subjects were excluded from the analysis due to gradient artifacts, errors caused by movements, or because the intensity of the MRI was inhomogeneous. The MRI data collection protocol for this study was approved by the NASA and University of Idaho institutional review boards and satisfied all local and international regulations for human subject research. All subjects provided informed written consent before participating in the study. Data were de-identified before being transferred to the University of Idaho for analysis.

### MRI acquisition and reformatting

T2-weighted axial spin-echo fat-suppressed MRI sequences were collected using a 3T system (Verio 3T; vB19; Siemens Healthineers, Erlangen, Germany) with 0.39 mm in plane isotropic pixel size (FOV 100 ×100), and 0.80 mm slice thickness and spacing. Additional sequence parameters included a 170° flip angle, 750 ms repetition time, 111 ms echo time, and 211 Hz/pixel bandwidth.

**Fig. 1:**
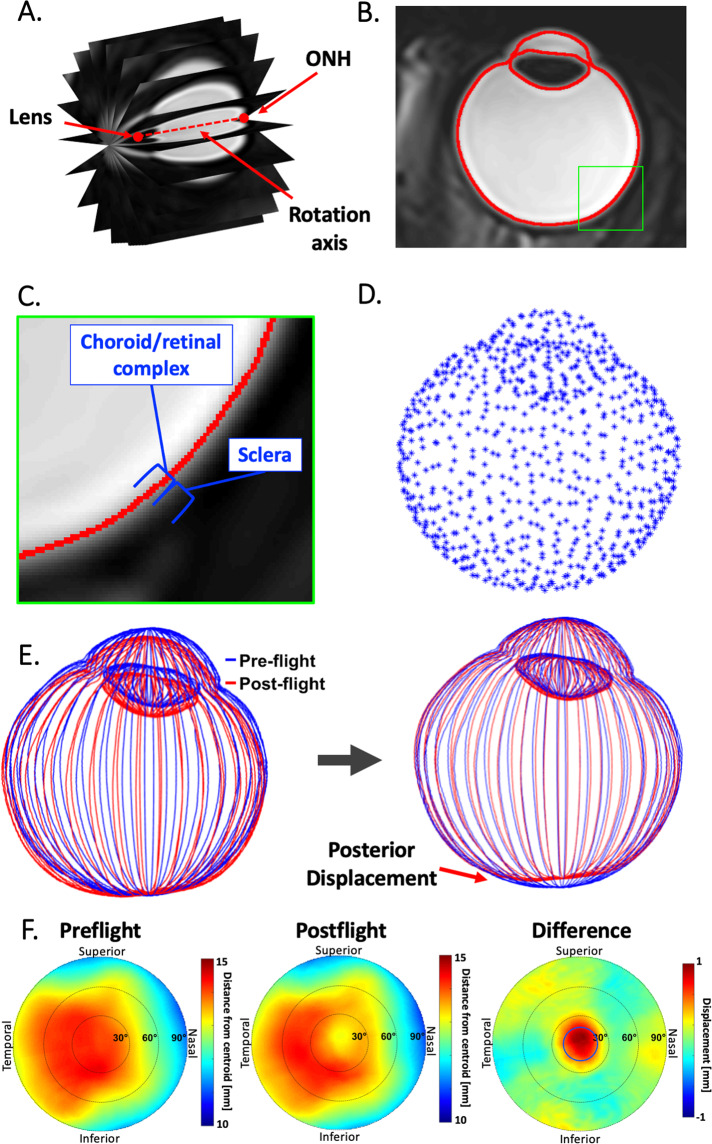
Methods for segmenting T2-weighted axial ocular MRI. **A** MR images were radially resliced, and **B** automatically segmented. **C** Zoomed in view of the posterior globe (green square on **B**), showing the sclera (hypointense region), the putative retinal/choroidal complex, and the boundary identified by our segmentation scheme (red curve). **D** 3D reconstructed downsampled point cloud, and **E** the registration of point clouds. **F** Example of pre- and postflight distance maps and the resulting differential displacement map for the posterior surface of one eye with notable globe flattening, within the 4 mm region of interest (blue circle) around the optic nerve head on the displacement map (colour figure online).

MRI scans of each orbit were radially resliced in Osirix (version 8.0.1, Pixmeo, Geneva, Switzerland) at 1-degree increments (180 slices) about a central rotational axis (Fig. 1A). This ensured consistent slice orientation orthogonal to the scleral surface. The rotation axis was defined by manual selection of the ONH centroid and lens centre, through which a view axis was aligned. The ipsilateral lens and ONH points were triangulated with the contralateral ONH point to manually define an axial plane. Each slice was exported as a 16-bit image with 512 rows and columns with minimal padding around the globe.

### Generation of point cloud

Three dimensional reconstructions of each globe were created and mapped to a common coordinate system using a multistep process in MATLAB (vers 2019a, Mathworks Corp. Natick, MA.). Global thresholding was used to segment the posterior globe (Fig. 1B). To account for variations in scan intensity, a histogram-based selection of background pixels was used to compute a threshold offset [12]. Each slice was cubically upsampled by a factor of four before applying a threshold value of 380 plus the predetermined offset for the respective scan. The initial threshold of 380 was chosen based on anatomical inspection of the resulting geometries with respect to the scleral margin and maximization of the total number of analyzed subjects. Previous MRI imaging studies have identified the sclera to be the markedly hypointense region on T2 images, with the retina and choroid presenting as a multilayered structure [13–15]. Based on comparison with these studies, our segmentation scheme identified a boundary near the exterior margin of the retinal/choroidal structure, i.e., slightly interior to the scleral/choroidal margin (Fig. 1C). We consider this boundary to be a reasonable representation of the inner surface of the ocular globe (see “Discussion”) and will thus refer to our segmentation procedure as “globe segmentation” going forward.

After thresholding, a flood fill operation was applied to preserve only the inner region of the globe. Next, the edge points of the globe were collected and transformed to 3D MRI coordinates. Every point cloud was downsampled using a box-grid filter (MATLAB, computer vision toolbox, pcdownsample) with a filter size of 0.5 to ensure that the points were uniformly spaced (Fig. 1D). The downsampled point clouds were then used in an alignment process. This process involved choosing a preflight point cloud as a baseline and registering each subsequent postflight point cloud using an iterative closest point algorithm (MATLAB, computer vision toolbox, pcregistericp) with 60 iterations (Fig. 1E).

### Generation of displacement map

Displacement maps were generated to visualize changes in the posterior globe. First, the centroid for each eye was defined by averaging the locations of all points in the downsampled preflight point cloud. Next, a set of coordinate axes was created from the normal vector to the manually defined axial slice and the axis joining the ONH to the centroid. Using these two vectors, a circumferential and meridional coordinate system was created as described by Grytz et al. [16]. The spherical coordinates of each point on the globe surface were then expressed in polar coordinates, i.e., circumferential and meridional angles were transformed into a polar angle and radial location, and radius was represented by a colour on the pre- and postflight distance maps. These points were interpolated onto a square grid to allow for pairwise comparison between the distance maps (Fig. 1F).

For each point on the ocular surface, a vector was defined from the centroid to that point, and the vector’s magnitude (length) was represented by a colour ranging from red to blue (Fig. 1F). A (postflight–preflight) displacement map of the posterior globe was created by subtracting these vector magnitudes (Fig. 1F), and displacements for surface points lying within a 4 mm radius of the ONH were quantified. A mean globe displacement value was computed based on the average displacement within this 4 mm radius. The associated volumetric change was determined by calculating the volume of a cylinder with a height equal to the mean displacement and a radius of 4 mm.

### Ocular measures

Axial lengths have been previously reported by Macias et al. [5] and are presented here to compare with globe flattening variables. In brief, axial length, i.e., the distance between the anterior surface of the cornea and the fovea, was measured before and after spaceflight using optical biometry (IOLMaster 500; Zeiss). Fundoscopy was performed on all crewmembers after flight as part of the standard postflight medical assessment. The presence of optic disc oedema was determined by reviewing fundus images. The modified Frisen grading system was used to describe the optic disc oedema, when present.

### Statistics

Statistical analyses were conducted using Stata/SE (v 16.0), setting two-tailed alpha to reject the null hypothesis at 0.05, with an emphasis on characterizing the observed effects and reporting statistical significance. Our experimental design was a mixed-factorial, with repeated observations nested within astronaut (left and right eye) and over time (several postflight time points), each representing a delta score from astronauts’ preflight values. All our outcomes were continuously scaled and were analyzed using Gaussian-based maximum likelihood mixed-effects modelling that included 2 random Y-intercepts for the nesting of left and right eye measurements within time period and to accommodate for the repeated-measures over time. We included a fixed-effects covariate parameter to adjust for each astronauts’ prior exposure to weightlessness (i.e., the number of previous flight days), and a priori contrast to compare delta scores at each time point (relative to preflight score) to zero. Statistical assumptions were tested before interpreting results, and 1 (out of 77) overly influential observation was eliminated from our evaluation of the novel volume displacement outcome because it had a standardized residual that exceeded ±2, which skewed overall distribution of residuals. We used Somers’ *d* measure of association and Bland & Altman plots with 95% levels of agreement references to evaluate the strength of association between the changes in ocular displacement and the changes in optical biometry, incorporating the nesting of observations within astronaut.

## Results

**Fig. 2:**
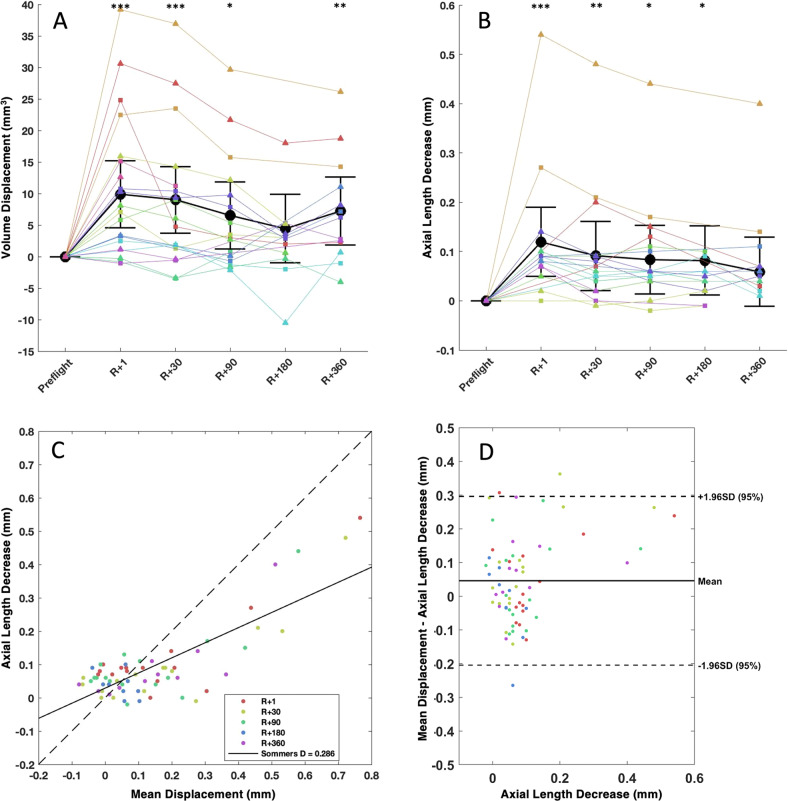
Plots showing volume displacement and ocular biometry changes after spaceflight. Plots showing pre- to postflight **A** MRI-assessed volume changes in the posterior ocular globe and **B** optical biometry-assessed axial length decreases at multiple time points after return to earth (R+; days) postflight. All changes are referenced to preflight values. Triangles and squares represent right and left eyes, respectively. Each subject is shown in a different colour, with yellow representing the subject diagnosed with grade 1 optic disc oedema. Black markers and error bars represent mean values with 95% confidence intervals and black stars indicate statistical significance from preflight baseline. Volume displacement within a 4 mm radius of the optic nerve head was averaged and compared with ocular axial length decreases (pre-post) as measured by ocular biometry using **C** correlation and **D** Bland–Altman plots. Note: **B** was created using data adapted from Macias et al. [5] *p < 0.05, **p < 0.01, ***p < 0.001 (colour figure online).

**Fig. 3:**
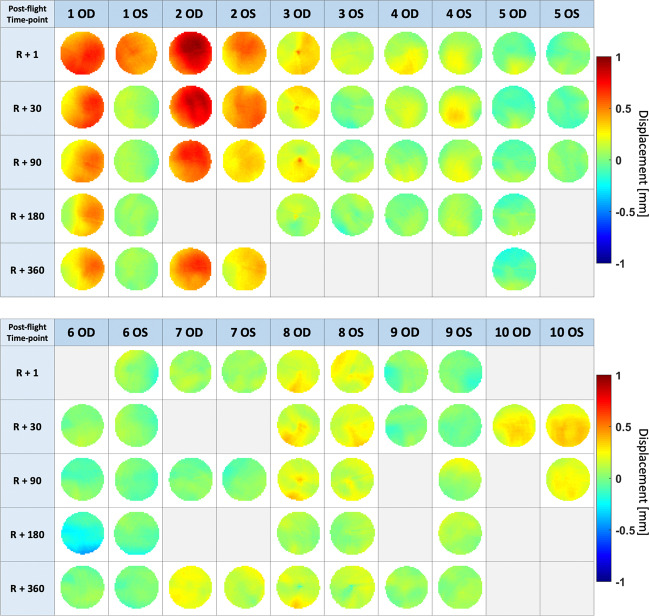
Summary of all globe displacement maps for each subject (within a 4 mm radius around the optic nerve head) at multiple time points (R + 1, R + 30, R + 90, R + 180, and R + 360 days). OS and OD refer to left and right eye respectively. Grey boxes indicate that data were not available for that time point. The subject with the most severe displacement (subject 2, OD) was clinically diagnosed with grade 1 optic disc oedema via fundus imaging.

Nearly all subjects exhibited some degree of globe flattening (Table 1). However, the degree of globe flattening was relatively small when compared to the average reported vitreous chamber volume of 4650 and 4969 mm^3^ in women and men, respectively [17], with an average volume displacement of 9.88 mm^3^ (95% CI 4.56–15.19 mm^3^, *p* < 0.001) measured at R + 1 (Table 1 and black line in Fig. 2A). During the postflight follow-up scans, mean volume displacement was 9.00 mm^3^ (95% CI 3.73–14.27 mm^3^, *p* = 0.001) at R + 30; 6.53 mm^3^ (95% CI 1.24–11.83 mm^3^, *p* < 0.05) at R + 90; 4.45 mm^3^ (95% CI −0.96 to 9.86 mm^3^, *p* = 0.12) at R + 180; and 7.21 mm^3^ (95% CI 1.82–12.60 mm^3^, *p* < 0.01) at R + 360. Notably, the subject with the greatest volume displacement (22.43 mm^3^ in the left eye and 39.16 mm^3^ right eye) is also presented with Frisén grade 1 optic disc oedema (Subject 2, Fig. 3).

**Table 1:** Mean and standard error of MRI-assessed posterior ocular globe changes from preflight (baseline), including change in volume, axial length (assessed by optical biometry), and mean displacement.

Parameter	R + 1	R + 30	R + 90	R + 180	R + 360
Volume decrease (mm^3^)	9.88 ± 5.31***	9.00 ± 5.27***	6.53 ± 5.29*	4.45 ± 5.41	7.21 ± 5.39**
Δ Ocular biometry (mm)	−0.12 ± 0.07***	−0.09 ± 0.07**	−0.08 ± 0.07*	−0.08 ± 0.07*	−0.06 ± 0.07
Mean displacement (mm)	0.20 ± 0.11***	0.18 ± 0.11***	0.13 ± 0.11*	0.09 ± 0.11	0.14 ± 0.10**
Number of subjects, eyes	9, 17	9, 18	10, 18	7, 12	7, 13

Key: *p < 0.05; **p < 0.01 ***p < 0.005.

The average decrease in axial length acquired by ocular biometry at R + 1 was 0.12 mm (95% CI 0.19–0.05 mm, *p* = 0.001), which partially recovered to 0.06 mm (95% CI 0.13–0.01 mm, *p* = 0.09) by R + 360 (Table 1). Volume displacement from MRI was used to generate the mean displacement parameter that could be compared with ocular biometry measurements. The average values of the mean displacement were 0.20 mm (95% CI 0.09–0.30 mm, *p* < 0.001) at R + 1; 0.18 mm (95% CI 0.07–0.28 mm, *p* = 0.001) at R + 30; 0.13 mm (95% CI 0.02–0.24 mm, *p* < 0.05) at R + 90; 0.09 mm (95% CI –0.02 to 0.20 mm, *p* = 0.12) at R + 180; and 0.14 mm (95% CI 0.04–0.25 mm, *p* < 0.01) at R + 360.

The mean difference between decrease in axial length from optical biometry and the mean displacement was 0.05 ± 0.12 mm. Linear correlation yielded an *R*^2^ of 0.62 and Sommers’ *d* of 0.286 (Fig. 2C). The Bland–Altman plot (Fig. 2D) shows agreement between these variables with most of the data points lying within the 95% confidence range.

## Discussion

Here we present an objective, MRI-based volumetric quantification of changes in the posterior globe of astronauts after ~6-month spaceflight missions. Our findings provide a quantitative analysis of individual posterior ocular globe volume displacement due to spaceflight, as well as postflight recovery of these changes, and our study lends support to previous subjective assessments of globe flattening in astronauts [1, 4, 7, 18]. Our findings suggest that pathological globe flattening is not widespread; however, there is a consistent trend of inward displacements of the peripapillary choroidal-scleral interface in most subjects after long-duration (~6-months) spaceflight. These displacements were small relative to the total volume of the globe and tended to be unidirectional within the region of interest, i.e., there were no instances where displacements occurred in both directions to create no net change within the 4 mm radius analyzed. The magnitude of inward displacement of the posterior globe decreased over time after return to Earth; however, on average, it had not returned to preflight baseline value by 1 year after spaceflight. Notably, one astronaut in our cohort with Frisén grade 1 optic disc oedema also had the greatest degree of posterior globe volume displacement. Our data are consistent with ocular biometry measurements which show that globe axial length did not return to preflight values by R + 360 (Fig. 2B) [5].

The longitudinal nature of this study and inclusion of multiple postflight recovery time points provides a unique quantitative assessment of spaceflight-induced globe flattening and its recovery, showing a clear association between the magnitude of globe volume displacement and duration of time after spaceflight. We speculate that displacement may have been more severe inflight but began recovering before the first postflight scan took place, occurring on average 4 days after return to Earth.

The majority of astronauts in this cohort showed inward displacements of the posterior globe at all time points with the exception of subject 6 who showed a significant outward displacement of the right eye at R + 180. This was the only observation out of 79 to show such a displacement. The cause of this displacement is not known but it was mainly isolated to the inferior aspect of the displacement map (Fig. 3, subject 6 OD) which differs from patterns observed in subjects with inward displacements. These scans were visually inspected for artifacts of which none were identified. It is possible that this subject had a natural variation in physiology at that time.

While volume displacement tended to decrease over time from R + 1 to R + 180, there was a marked increase in displacement between R + 180 and R + 360; further this increase differed from the observed decrease in axial length as measured by ocular biometry. We are not aware of any event between R + 180 and R + 360 that would explain the observed increase in displacement from R + 180 and R + 360. We point out that it was not possible to collect data for all astronauts at both R + 180 and R + 360, and that the set of time points/astronauts for which ocular biometry measurements were “missing” differed from the time points/astronauts for “missing” MRI. This missing data might explain the discrepancy between MRI and ocular biometry, and the apparent reversal of the volume displacement trend after R + 180. In addition, there was one astronaut who had a notable negative change in globe volume displacement in one eye at R + 180, which may have resulted in an overestimation of the predicted recovery at R + 180 and an exaggeration of the apparent increase in displacement between R + 180 and R + 360. Despite this R + 360 observation, the overall dataset demonstrates a spaceflight-induced increase in globe flattening that slowly recovers over a year.

It is important to note that our segmentation methods detect an anatomic boundary near the choroidal/scleral interface, and thus the displacement we compute could be due to morphometric changes other than scleral flattening, i.e., our displacement parameter combines globe flattening with choroidal swelling. However, the average mean displacement measured in this study far exceeded the average combined increase in total retinal and choroidal thickness identified using optical coherence tomography by Macias et al. [5]. Moreover, spaceflight induced total retinal and choroid thickening on average recover to preflight values by 90 days after landing [5]. Thus, changes in the choroid cannot explain the magnitude of the displacements that we observed in most subjects, and throughout we use the term “globe flattening” to describe the morphological changes that we have observed.

Several studies have reported clinical ocular examinations of astronauts who participated in long-duration spaceflight. Mader et al. subjectively identified globe flattening and optic disc oedema in 5 of the 7 astronauts they assessed, and identified thickening of the retinal nerve fibre layer in 6 of 7 astronauts [4]. In 3 of these 7 astronauts, globe flattening persisted for at least 7 years following spaceflight [19]. A 2017 case report described an astronaut who developed asymmetric optic disc oedema during spaceflight, which remained unresolved 630 days after flight [18]. Optic disc oedema was also identified in an astronaut cohort similar to the cohort assessed in the present study, and the reported recovery pattern for the optic disc oedema was similar to that of the recovery of globe flattening presented here [5]. Subjective, MRI-based clinical reporting by Kramer et al. identified globe flattening in 7 of 27 astronauts [1].

Semi-automated MRI-based mapping of the optic globe has been performed previously in several studies (Table 2), with each study producing different parametrizations. These parameterizations require development of a coordinate system that depends on anatomical reference points, typically accomplished by image registration and definition of an anatomical axis. In this work, we used an iterative closest point algorithm with a least squares metric to minimize the differences between matching point clouds. This is considered to be a conservative approach because all points are weighted equally. Performing registration after segmentation helps align the unanchored globe in MRI images, but could introduce error from segmentation artifacts and loss of volumetric information. The fairness of the registration depends on point cloud uniformity, which was enforced with a box-grid filter. Our segmentation was aided by radially reslicing the MRI scan of the orbit (Fig. 1A) to obtain consistent intervoxel averaging of the edge of the globe. This highly automated registration method, in combination with the advanced multi-time point study design, adds rigour to the current study. Alperin et al. applied a mapping technique to measure globe flatness in IIH patients. When compared to a control group, IIH patients showed a globe flatness index increase of 0.02 [8], corresponding to a linear displacement of ~0.24 mm, similar to the average mean displacement we found in astronauts.

**Table 2:** Comparison of various MRI-based methods used to quantify optic globe structure.

Method	MRI protocol	Key finding and measures	Citation
*Segmentation* Expectation-maximization *Parameterization* Axial plane matching with rigid linear MRI registration; Orthogonal coordinate system with respect to axis formed by lens centre to globe centre of mass.	1.5/3T T2 3D CISS: 0.6 mm in plane isotropic; slice thickness, 0.6 mm; TR, 6.35/5.42 ms; TE, 2.82/2.43 ms; flip angle, 47/34°; pixel bandwidth, 560/650 Hz/pixel	Posterior globe is altered in IIH * 2D cartesian deformation map and corresponding measures: nerve protrusion (NP), globe flattening (GF), maximum deformation (MD) * Control, IIH mean ± SD measures, respectively: NP 0.96 ± 0.013, 0.91 ± 0.028, *p* = .00002 GF 0.93 ± 0.020, 0.91 ± 0.022, *p* = .0035 MD 0.93 ± 0.021, 0.88 ± 0.027, *p* = .00002	Alperin et al. [8]
*Segmentation* Flood fill and morphology *Parameterization* Conic projection based on conic axis defined by lens centre to centre of vitreous body.	T1 3D inversion recovery turbo gradient echo: 0.5 mm in plane isotropic (FOV 40 × 46 mm); slice thickness, 1 mm; TR, 2.5 ms; TE, 4.55 ms; flip angle, 16°; inversion time, 1280 ms;	Retinal distance map can be quantified by MRI * MRI vs optical biometry axial length mean ± SD difference: 0.08 ± 0.23 mm *p* = 0.01 * MRI retinal map reliability: SD = 0.11 mm (4 subjects, scanned twice) * Does not account for patient orientation changes	Beenakker et al. [23]
*Segmentation* Flood fill followed by spherical mesh shrink-wrap and local averaging. *Parameterization* Circumferential w.r.t axial length	T2 half-acquisition turbo spin-echo sequence: 0.5 mm place isotropic (FOV, 256 × 256 mm); 1 mm slice thickness; TR, 1240 ms; TE, 124 ms; flip angle, 150°; 6 averages; 4/8 partial-phase acquisition	Axial length can be quantified by MRI * Circumferential colour-coding with respect to axial length * Axial length intersession repeatability (one subject, 10 scans) mean ± SD: Right eye = 23.78 ± 0.27 mm Left = 24.41 ± 0.52 mm	Singh et al. [24]
*Segmentation* Intensity offset global thresholding *Parameterization* Iterative closest point registration; circumferential and meridional coordinate system.	T2 axial spin-echo fat suppressed MRI: 0.390 mm in plane isotropic (FOV, 100 × 100); 0.800 mm slice thickness and spacing; TR, 750 ms; TE 111 ms; flip anlge, 170°; pixel bandwidth, 211 Hz/pixel.	Retinal changes unresolved after spaceflight. * 2D polar displacement map * Mean displacement moderately correlated with change in ocular biometry axial length: R^2^ = 0.624. * Mean difference between change in ocular biometry axial length and mean displacement: 0.045 ± 0.124 mm. * Not dependent on patient orientation	Current study

*CISS* constructive interference in steady state, *TR* repetition time, *TE* echo time, *FOV* field of view.

Ocular biometric measurements are the gold standard for measuring anatomical characteristics of the eye [20], including the axial length. In the astronaut population, the difference between pre and postflight axial length provides a single value to quantify globe flattening. Axial length is a clinically accepted measurement against which our displacements can be compared, although the displacement parameter computed in our study is based on a different optical axis than axial length. Specifically, while axial length is measured along the optical axis of the eye (cornea to fovea), our displacement quantifies changes along an axis between the centre of the lens and the location of the optic nerve head (roughly 4 mm from the fovea). Although this is slightly different to the axial length parameter, it does permit a valid comparison of changes in posterior globe shape.

The mechanisms underlying globe flattening are likely multifactorial and complex, e.g., orbital pressure may be altered in microgravity, yet this has not been investigated [21]. Since only a subset of astronauts develops clinically relevant globe flattening, there are likely individual-specific risk factors for globe flattening.

IIH is often discussed in the context of SANS because optic disc oedema, optic nerve sheath distention, and globe flattening are observed in both disorders. In IIH, these clinical features have been attributed to elevated ICP, and thus a decrease or reversal of the TLPD [9, 22]. However, astronauts do not exhibit many of the other symptoms of IIH, including chronic headache, and diplopia, and unlike the bilateral globe flattening associated with IIH, globe flattening associated with SANS can be asymmetric [4]. In the present study, the two subjects with the most significant globe flattening had flattening present in both eyes, although the degree of flattening was greater in the right eye for those subjects (Fig. 2A). While the duration of exposure for astronauts is still relatively short, it may take many years for these types of symptoms to develop in patients with IIH. Further research is needed to elucidate any similarities in the aetiologies of SANS and IIH.

Some limitations exist in this study, including the small number of astronaut subjects studied. The methods described here do not account natural variability in human physiology over time. The inherent challenges in MRI acquisition and segmentation can lead to artifacts in images of the cornea and lens, requiring subjective assessment of geometric agreement between point clouds. Despite these limitations, we have shown that our technique can consistently detect and quantify volume displacement. Although MRI techniques can be used to examine orbital anatomy, scanning is not feasible during spaceflight, restricting these types of studies to pre- and postflight assessment. Recovery likely begins immediately after return to Earth, and if so, our first MRI scan around 4 days after landing may not fully reflect inflight values.

The goal of this study was to develop and apply an automated non-invasive method to prospectively quantify posterior globe flattening in long-duration spaceflight astronauts, as well as the postflight recovery profile. A novel technique was developed and used to quantify posterior globe volume displacement from MRI scans in ten astronauts after their respective ~6-month spaceflight missions, and at five recovery time points after spaceflight. The greatest degree of globe displacement was detected in our first postflight scan, and values gradually recovered with time after flight but were only partially resolved within 1 year. Further work is needed to fully understand the aetiology of SANS, including the cause(s) of globe flattening. The application of the method in healthy subjects over time will help define the resolution of the method, and the application of the method in more astronauts who have participated in spaceflights of different durations will help identify the relationship between globe flattening and spaceflight duration. Coupling future research MRIs with other ocular measures will help us understand future cases of outward globe displacement, should they arise.

## Summary

### What was known before

- Long-duration spaceflight is associated with euro-ocular changes that affect visual acuity.
- These changes can include choroidal folds, optic disc oedema, cotton wool spots, and hyperopic shifts, and these symptoms are associated with spaceflight associated neuro-ocular syndrome (SANS).

### What this study adds

- Long-duration spaceflight is associated with flattening of the posterior globe.
- Posterior globe flattening persists beyond 1 year after return to Earth.
